# Factors associated with weight gain in pre- and post-menopausal women receiving adjuvant endocrine therapy for breast cancer

**DOI:** 10.1007/s11764-023-01408-y

**Published:** 2023-06-01

**Authors:** Anna-Carson Rimer Uhelski, Amanda L. Blackford, Jennifer Y. Sheng, Claire Snyder, Jennifer Lehman, Kala Visvanathan, David Lim, Vered Stearns, Karen Lisa Smith

**Affiliations:** 1 Johns Hopkins Department of Medicine, Johns Hopkins University School of Medicine, Baltimore, MD USA; 2 Present Address: Hematology/Oncology Fellowship Program, Vanderbilt University Medical Center, Nashville, TN USA; 3 Division of Biostatistics and Bioinformatics, Johns Hopkins Sidney Kimmel Comprehensive Cancer Center, Baltimore, MD USA; 4 Johns Hopkins Women’s Malignancies Disease Group, Johns Hopkins University School of Medicine, Baltimore, MD USA; 5 Department of Health Policy and Management, Johns Hopkins Bloomberg School of Public Health, Baltimore, MD USA; 6 Department of Oncology, Johns Hopkins University School of Medicine, Baltimore, MD USA; 7 Division of Cancer Epidemiology, Johns Hopkins Bloomberg School of Public Health, Baltimore, MD USA; 8 Present Address: Division of Statistics, Collaborative Inc., WCG, Washington, DC USA; 9 Sidney Kimmel Comprehensive Cancer Center, Johns Hopkins School of Medicine, Under Armour Breast Health Innovation Center, The Skip Viragh Outpatient Cancer, Building 201 North Broadway Viragh 10th floor, Room 10291, Baltimore, MD 21287 USA; 10 Present Address: AstraZeneca, Gaithersburg, MD USA

**Keywords:** Breast cancer, Adjuvant endocrine therapy, Weight gain, Patient-reported outcomes, Obesity

## Abstract

**Purpose:**

Weight gain after breast cancer poses health risks. We aimed to identify factors associated with weight gain during adjuvant endocrine therapy (AET).

**Methods:**

Women initiating AET enrolled in a prospective cohort. Participants completed FACT-ES plus PROMIS pain interference, depression, anxiety, fatigue, sleep disturbance and physical function measures at baseline, 3, 6, 12, 24, 36, 48 and 60 months. Treatment-emergent symptoms were defined as changes in scores in the direction indicative of worsening symptoms that exceeded the minimal important difference at 3 and/or 6 months compared to baseline. We used logistic regression to evaluate associations of clinicodemographic features and treatment-emergent symptoms with clinically significant weight gain over 60 months (defined as ≥ 5% compared to baseline) in pre- and post-menopausal participants.

**Results:**

Of 309 participants, 99 (32%) were pre-menopausal. The 60 months cumulative incidence of clinically significant weight gain was greater in pre- than post-menopausal participants (67% vs 43%, *p* < 0.001). Among pre-menopausal participants, treatment-emergent pain interference (OR 2.49), aromatase inhibitor receipt (OR 2.8), mastectomy, (OR 2.06) and White race (OR 7.13) were associated with weight gain. Among post-menopausal participants, treatment-emergent endocrine symptoms (OR 2.86), higher stage (OR 2.25) and White race (OR 2.29) were associated with weight gain while treatment-emergent physical function decline (OR 0.30) was associated with lower likelihood of weight gain.

**Conclusions:**

Weight gain during AET is common, especially for pre-menopausal women. Clinicodemographic features and early treatment-emergent symptoms may identify at risk individuals.

**Implications for cancer survivors:**

Patients at risk for weight gain can be identified early during AET.

**Clinical trials.gov identifier:**

NCT01937052, registered September 3, 2013.

**Supplementary information:**

The online version contains supplementary material available at 10.1007/s11764-023-01408-y.

## Introduction

Obesity, defined as body mass index (BMI) > 30 kg/m^2^, is common among patients with breast cancer, both at the time of diagnosis and afterwards [1–3]. Excess weight is an established risk factor for developing breast cancer in post-menopausal women [4, 5]. Weight gain *after* breast cancer diagnosis is reported in up to 90% of women, with many studies suggesting pre-menopausal patients are at particular risk for post-diagnosis weight gain [2, 5–19]. Weight gain after breast cancer can pose serious health consequences and can cause psychological distress and poor quality of life [10, 20–29]. Breast cancer survivors with obesity or who gain clinically significant weight face higher risks of recurrence and mortality [4, 10, 26–28, 30–36].

The role of breast cancer therapy in post-diagnosis weight gain is poorly defined. Multiple studies suggest receipt of adjuvant chemotherapy is associated with weight gain; however, whether findings with regard to the association of adjuvant endocrine therapy (AET) and weight gain have been inconsistent [2, 6–9, 11–15, 17, 18, 28, 37–43]. Over two thirds of breast cancers are hormone receptor (HR)-positive and at least 5 years of AET reduces recurrence and death in patients with HR-positive disease [39, 44–46]. The type of AET administered often differs according to menopausal status, with post-menopausal patients typically receiving an aromatase inhibitor (AI) and pre-menopausal patients receiving either tamoxifen, ovarian function suppression (OFS) with tamoxifen, or OFS with an AI [47–49]. While some studies have reported differences in the likelihood of weight gain according to type of AET, others have not, and risk factors for weight gain during AET are not well defined [2, 6–9, 11–14].

AET is frequently associated with side effects such as musculoskeletal discomfort, fatigue, sleep disturbance, mood changes, anxiety, and endocrine symptoms including hot flashes and vaginal dryness [29, 47, 50–64]. While each of these symptoms may occur with any type of AET, the side effect profile differs somewhat by type of AET, as exemplified by more frequent musculoskeletal symptoms with AIs compared to tamoxifen [11, 50, 65–68]. Treatment-emergent side effects with AET often develop soon after initiation [69–71]. AET and its side effects can affect quality of life, physical activity, sleep, and mood, all of which have been associated with weight gain after breast cancer diagnosis [6, 11, 29, 51–58]. Unfortunately, side effects during AET are not always detected during routine clinical care. The use of patient-reported outcomes (PRO), health assessments from patients without interpretation by a member of the clinical team, typically collected via questionnaires, enhances symptom detection [72–74]. To date, evaluation of the association between patient-reported symptoms and weight gain during AET has been limited [6, 11, 29, 38, 51–58].

We report here results of a secondary analysis from a prospective clinic-based cohort of patients with HR-positive breast cancer receiving AET who completed serial PRO symptom assessments over 60 months. Our analysis sheds further light on the association of AET with post-diagnosis weight gain and on risk factors, including patient-reported symptoms, for weight gain during AET. We describe weight trajectories and evaluate associations between clinicodemographic factors and symptoms emerging during the first 6 months of AET with clinically significant weight gain throughout the course of AET. Given prior literature suggesting patterns of weight gain during AET may vary by menopausal status, known differences in the side effect profiles of tamoxifen and AIs and differences in prescribing patterns for these agents according to menopausal status, we conducted analyses separately for pre- and post-menopausal participants[2, 47–50]. For this secondary analysis, we hypothesized that weight gain during AET is associated with patient-reported treatment-emergent symptoms and that it differs according to menopausal status. Identification of factors associated with weight gain early in the course of AET may allow the opportunity for targeted interventions to prevent weight gain in at-risk breast cancer survivors.

## Methods

### Study population

Women with HR-positive stage 0-III breast cancer initiating AET with either tamoxifen or an AI were enrolled in an IRB-approved prospective observational clinic-based cohort from March 2012 through December 2016 (ClinicalTrials.gov Identifier: NCT01937052, registered September 3, 2013). The cohort was comprised of a convenience sample of female patients with breast cancer age ≥ 18 years who were seen in medical oncology clinics at Johns Hopkins clinical sites. Potential participants were identified by provider referral or by screening clinic schedules. The type of AET prescribed was at the discretion of the treating provider. In addition to tamoxifen or an AI, pre-menopausal participants could receive concurrent OFS. Participants could enroll when they first started AET or if they were switching from one AET agent to another. Participants were followed until the final PRO questionnaire or the last clinical encounter prior to the date the database was locked (May 15, 2020), whichever was longer. Demographics, cancer characteristics, treatment and menopausal status at diagnosis (classified as pre-menopausal or post-menopausal) were obtained via review of the electronic health record (EHR). Participants were eligible for inclusion in this secondary analysis if baseline weight and at least one follow-up weight were documented in the EHR.

### Patient-reported outcomes

PRO measures were administered electronically using the online PatientViewpoint interface [75–77]. Questionnaires were administered at baseline and 3, 6, 12, 24, 36, 48 and 60 months later. Measures included in this analysis are the Patient-Reported Outcomes Measurement Information System (PROMIS) Version 1.0 short forms for pain interference, depression, anxiety, fatigue, sleep disturbance and physical function plus the Endocrine Symptom Subscale of the Functional Assessment of Cancer Therapy (FACT-ES) [59, 78–80]. We defined treatment-emergent symptoms as changes in PRO scores compared to baseline during the first 6 months of AET (i.e. at the 3 and/or 6-month time points) that met or exceeded the minimal important difference (MID) for each measure in the direction indicative of worsening symptoms. The MID is the change in score on a PRO measure that represents the smallest difference perceived by patients as beneficial or harmful and that would impact clinical management [81]. PROMIS measures are scored with a T-score metric for which 50 is the population mean and 10 is the population standard deviation (SD). A higher score indicates more of the outcome measured. PROMIS measures have been validated in patients with early stage cancer with a MID of 3–5 points [78–80, 82, 83]. We considered the mid-point of this range, 4 points, as the MID for the PROMIS questionnaires in this analysis. Scores on the Endocrine Symptom Subscale of the FACT-ES range from 0–76, with lower scores indicative of worse endocrine symptoms. We used 0.5 SD to define a medium effect size as a conservative estimate of the MID for the Endocrine Symptom Subscale of the FACT-ES based on the distribution-based, Effect Size Method of identifying a MID for a PRO measure. In prior studies using the Endocrine Symptom Subscale of the FACT-ES in patients with early stage breast cancer, the reported mean (SD) was 59 (9.7), thus we halved the SD and rounded this estimate to 5 points to identify the MID for this analysis [59, 81, 84].

### Weight and body mass index

Weight (in kg) and BMI were obtained from the EHR using measurements assessed during routine clinic visits. BMI was categorized according to the World Health Organization categorization as underweight (< 18.5 kg/m^2^), normal weight (18.5 kg/m^2^ – 24.9 kg/m^2^), overweight (25 kg/m^2^ – 29.9 kg/m^2^), or obese (> 30 kg/m^2^) [3, 85]. Per study protocol, the heaviest weight and BMI documented in the medical record within ± 30 days of questionnaire collection was used in analysis. If a patient did not have a weight or BMI documented in the medical record within ± 30 days of each questionnaire time point, the weight value was missing for that time point. Consistent with the threshold used in many prior studies, we defined clinically significant weight gain as weight gain of ≥ 5% compared to baseline, a weight change that has been associated with increased all-cause mortality after breast cancer [10, 11, 27, 28, 42].

### Statistical analysis

Participant demographics, cancer characteristics, cancer treatment, weight, BMI and PRO scores are presented with descriptive statistics including mean (SD), median (range) and proportions. Clinically significant weight gain status was treated as a dichotomous variable indicating whether patients experienced ≥ 5% weight gain from baseline at each time point. Differential changes in weight gain status over time between the pre- and post-menopausal groups were assessed with a mixed effects logistic regression model and corresponding interaction terms. Differences in the proportion of patients with treatment-emergent symptoms according to menopause status were assessed with Fisher’s exact tests.

To estimate the incidence of weight gain during follow-up, time to weight gain was calculated as the time from baseline to either the first study time point where patients experienced ≥ 5% weight gain or last follow-up visit if they did not gain ≥ 5% weight. The cumulative incidence of weight gain was estimated using the Kaplan–Meier method.

We evaluated the association of clinicodemographic factors and symptoms emerging during the first 6 months of AET with clinically significant weight gain using univariate and multivariate logistic regression modeling with generalized estimating equations (GEE) to account for the longitudinal design and correlation of repeated measures within a participant. Univariate associations were estimated separately according to menopause status. Factors differentially associated with weight gain by menopause status were explored using interaction terms in the models. Multivariate models were estimated separately for pre- and post-menopausal participants using a forward and backward stepwise selection approach based on Quasi-likelihood under the Independence model Criterion (QIC)[86]. The models with the lowest QIC were selected. Non-time dependent demographic variables included in the models were age at enrollment, race (White versus Black/Other) and neighborhood poverty (NP) level. NP level was defined as the percentage of persons living in a zip code with a family income below the federal poverty line (based on United States 2010 census data); this was used as a surrogate for socioeconomic status (SES) with NP level > 15% considered an indicator of low SES [87]. Non-time dependent clinical variables included baseline PRO scores, baseline BMI category (overweight/obese compared to normal/underweight), prior radiation (yes/no), prior chemotherapy (yes/no), mastectomy (yes/no), stage (classified as a continuous variable), number of concomitant medications at baseline (self-reported), and type of AET (AI or tamoxifen). Since only 4 pre-menopausal participants received an AI, we conducted a sensitivity analysis re-running the model selection for the pre-menopausal participants excluding the variable for type of AET. There was no formal hypothesis testing nor sample size considerations for this study. The findings presented here are for descriptive purposes and no adjustments for multiple comparisons were made. Analyses were performed with R version 4.0.3 [88].

## Results

### Participant characteristics

Of the 321 participants in the overall cohort, baseline and at least one follow-up weight were available for 309 participants, who were therefore included in this secondary analysis. The 12 participants excluded from this analysis due to missing weight were generally similar to the 309 who were included (Supplemental Table 1). Among participants included in this analysis, 263 (85.1%) had stage I-II disease, 99 (32%) were pre-menopausal, and 259 (83.8%) were White. Prior to initiating AET, 140 (45.3%) underwent mastectomy, 205 (66.3%) received radiation and 86 (28%) received chemotherapy. More pre-menopausal participants than post-menopausal participants received prior chemotherapy (33.7% versus 25.4%). Overall, 132 (42.7%) participants initiated tamoxifen and 177 (57.3%) initiated an AI. The type of AET differed according to menopausal status. Ninety-five (96%) and 4 (4%) pre-menopausal participants initiated tamoxifen and AI, respectively. In contrast, 37 (17.6%) and 173 (82.4%) post-menopausal participants initiated tamoxifen and AI, respectively. Seventeen (17.2%) pre-menopausal participants received OFS. Only 5 participants enrolled upon switching form one type of AET to another. Median follow-up was 56 months (Table 1).

**Table 1:** Baseline Characteristics of Study Population According to Menopausal Status

Characteristic	Pre-Menopausal N = 99	Post-Menopausal N = 210	All Participants N = 309
Mean Age in years (SD)	50.2 (6.6)	68.3 (7.4)	62.5 (11.1)
Race—N (%)			
Black	9 (9.1)	23 (11)	32 (10.4)
White	80 (80.8)	179 (85.2)	259 (83.8)
Other	10 (10.1)	8 (3.8)	18 (5.8)
Endocrine Therapy—N (%)			
Tamoxifen ± OFS^a^	95 (96)	37 (17.6)	132 (42.7)
AI ± OFS^a^	4 (4)	173 (82.4)	177 (57.3)
Enrolled upon switching from one endocrine therapy to another	0 (0)	5 (2.3)	5 (1.6)
Stage—N (%)			
0	3 (3)	22 (10.5)	25 (8.1)
I	61 (61.6)	125 (59.5)	186 (60.2)
II	31 (31.3)	46 (21.9)	77 (24.9)
III	4 (4)	17 (8.1)	21 (6.8)
ER-positive—N (%)	99 (100)	210 (100)	309 (100)
PR-Positive—N (%)^b^	93 (94.9)	179 (85.2)	272 (88.3)
HER-2-Positive—N (%)^b^	7 (7.3)	18 (9.6)	25 (8.8)
Mastectomy—N (%)	59 (59.6)	81 (38.6)	140 (45.3)
Radiation—N (%)	130 (69.1)	75 (62)	205 (66.3)
Chemotherapy—N (%)^b^	33 (33.7)	53 (25.4)	86 (28)
Mean (SD) Baseline BMI (kg/m2)	25.9 (6.0)	28.2 (5.6)	27.5 (5.8)
Obese – N (%)	20 (20.2)	80 (38.1)	100 (32.3)
Overweight – N (%)	27 (27.2)	64 (30.5)	91 (29.4)
Median Number of Concomitant Medications (Range)	3 (0–16)	5 (0–29)	4 (0–29)
NP Rate^b,c^ > 15%—N (%)	18 (18.2)	24 (11.5)	42 (13.7)
Median Follow-up time in Months (Range)	58.1 (12.2–87.7)	54.7 (6.9–87.3)	56.0 (6.9–87.7)

SD = Standard Deviation, OFS = Ovarian Function Suppression, ER = estrogen receptor, PR = progesterone receptor, HER2 = Human Epidermal Growth Factor Receptor-2, BMI = body mass index, NP = neighborhood poverty

a. Seventeen pre-menopausal participants received OFS (17.2%). Of these, one received an AI and 16 received tamoxifen. No post-menopausal participants received OFS

b. Denominator for percentages was based on the number of known assessments. PR status was missing for one pre-menopausal participant. HER2 status was missing for 3 pre-menopausal and 22 post-menopausal participants. Prior chemotherapy status was missing for one pre-menopausal and one post-menopausal participant. NP rate was missing for two post-menopausal participants

c. NP rate is the percentage of persons living in a zip code with a family income below the federal poverty line based on United States census data

Mean (SD) BMI at baseline for the entire study population included in this analysis was 27.5 kg/m^2^ (5.8). Baseline BMI was in the overweight category for 91 (29.4%) participants and in the obese category for 100 (32.2%) participants (Table 1). The distribution of baseline BMI categories differed by menopausal status with 47 (47.4%) pre-menopausal compared to 144 (68.6%) post-menopausal participants having a baseline BMI ≥ 25 kg/m^2^ (*p* < 0.001) (Supplemental Table 2). At baseline, 158 (61%) White participants, 24 (75%) Black participants and 9 (50%) participants of other race were overweight or obese.

### Weight gain

**Fig. 1:**
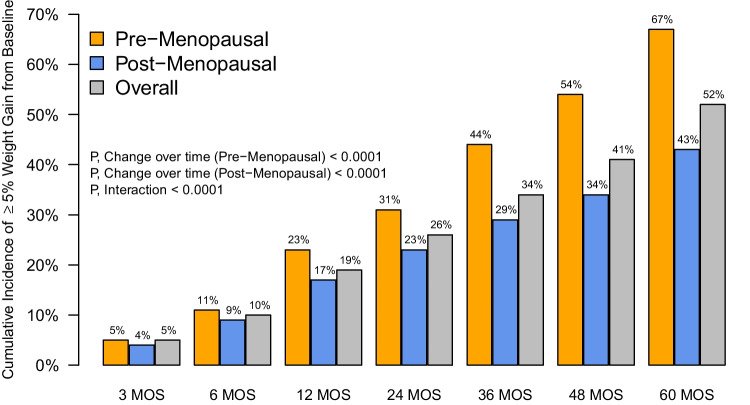
Cumulative Incidence of Clinically Significant Weight Gain at Each Time Point by Menopausal Status**.** Bars indicate the cumulative incidence of experiencing ≥ 5% weight gain compared to baseline at each time point. Gray bars indicate the overall population; blue bars indicate the post-menopausal participants; and orange bars indicate the pre-menopausal participants. Abbreviations: MOS = Months

Overall, 121 (39.2%) participants experienced ≥ 5% weight gain compared to baseline on at least one follow-up assessment. Clinically significant weight gain frequently occurred early, with a cumulative incidence of 19% experiencing ≥ 5% weight gain by 12 months after AET initiation, and continued over time, with a cumulative incidence of 52% experiencing ≥ 5% weight gain by 60 months after AET initiation. The proportions of pre-menopausal and post-menopausal participants who gained ≥ 5% weight compared to baseline both increased over the course of follow-up (p-values evaluating weight gain status over time within pre-menopausal and post-menopausal groups both < 0.001). However, compared to post-menopausal participants, significantly more pre-menopausal participants gained ≥ 5% weight over the course of follow-up (interaction p-value < 0.001). By 60 months, the cumulative incidence of ≥ 5% weight gain compared to baseline was 67% for pre-menopausal and 43% for post-menopausal participants (Fig. 1). Mean (SD) weight gain by 60 months for pre- and post-menopausal participants was 3.5 (10.4) kg and 0.6 (6.5) kg, respectively. The number of patients with missing weight measurements increased over time for both pre- and post-menopausal participants (Supplemental Table 3).

### Scores on PRO measures

**Fig. 2:**
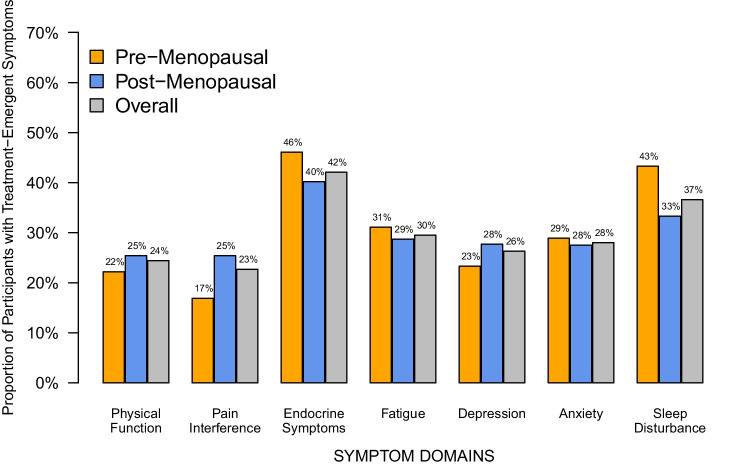
Proportions of Participants with Treatment-Emergent Symptoms by Menopausal Status**.** Bars indicate the proportion of participants who experienced treatment-emergent symptoms, defined as changes in PRO scores compared to baseline at the 3 and/or 6-month time points that met or exceeded the MID in the direction indicative of worsening symptoms; Gray bars indicate the overall population; blue bars indicate the post-menopausal participants; and orange bars indicate the pre-menopausal participants. Abbreviations: PRO = Patient-Reported Outcome, MID = Minimal Important Difference

Overall, mean scores on each PRO measure at baseline, 3 and 6 months were within one SD of population means. The number of completed questionnaires declined through follow-up (Supplemental Table 4). Treatment-emergent symptoms, defined as changes in PRO scores compared to baseline at the 3 and/or 6-month time points that met or exceeded the MID in the direction indicative of worsening symptoms, were common, each occurring in 23%-42% of the overall study population (Fig. 2). Endocrine symptoms (42%) and sleep disturbance (37%) were the most common treatment-emergent symptoms, followed by fatigue (30%), anxiety (28%) and depression (26%). There were no statistically significant differences in the proportions of pre-menopausal and post-menopausal participants who experienced each type of treatment-emergent symptom (Fisher’s exact test p-values all > 0.05).

### Association of treatment-emergent symptoms and clinicodemographic factors with weight gain in pre-menopausal and post-menopausal participants

Univariate and multivariate logistic regression analyses of factors associated with ≥ 5% weight gain compared to baseline are shown in Table 2. In univariate analyses among pre-menopausal participants, prior mastectomy (Odds Ratio [OR] 2.23, 95% Confidence Interval (CI) 1.00–4.96, *p* = 0.05) and White race (OR 6.45, 95% CI 1.18–35.25, *p* = 0.03) were associated with weight gain while participants with obesity at baseline were less likely to gain ≥ 5% weight compared to baseline than those who were normal weight or underweight at baseline (OR 0.30, 95% CI 0.10–0.93, *p* = 0.04). In univariate analysis among post-menopausal participants, higher tumor stage (OR 1.84, 95% CI 1.27–2.67, *p* = 0.001) and treatment-emergent endocrine symptoms (OR 2.05, 95% CI 1.10–3.82, *p* = 0.02) were associated with greater likelihood of ≥ 5% weight gain while treatment-emergent decline in physical function was associated with lower likelihood of ≥ 5% weight gain (OR 0.36, 95% CI 0.16–0.83, *p* = 0.02). The associations of treatment-emergent change in physical function and treatment-emergent pain interference with weight gain status differed by menopausal status (interaction p-values both ≤ 0.05).

**Table 2:** Univariate and Multiple Logistic Regression Analyses of Factors Associated with ≥ 5% Weight Gain by Menopausal Status

Univariate Logistic Regression Model			
Variable	Odds Ratio (OR)^a^ (95% confidence interval, p-value)		Interaction p-value^b^
	Pre-menopausal	Post-menopausal	
Age (in years)	0.98 (0.91–1.05, 0.55)	0.98 (0.93–1.02, 0.32)	0.96
Radiation	0.56 (0.26–1.20, 0.14)	1.17 (0.61–2.27, 0.63)	0.15
Chemotherapy	1.63 (0.76–3.49, 0.21)	1.80 (0.92–3.54, 0.09)	0.75
Mastectomy	2.23 (1.00–4.96, 0.05)	1.12 (0.59–2.09, 0.74)	0.21
Higher Stage	1.00 (0.54–1.85, 0.99)	1.84 (1.27–2.67, 0.001)	0.08
AET (AI vs Tam)	4.85 (0.73–32.02, 0.10)	2.09 (0.77–5.64, 0.15)	0.51
Race (White vs Black/Other)	6.45 (1.18–35.25, 0.03)	2.03 (0.76–5.45, 0.16)	0.41
NP rate^c^	0.89 (0.62–1.29, 0.54)	0.89 (0.68–1.17, 0.40)	0.9
Baseline BMI^b^ (25–29.9 kg/m^2^ vs < 25 kg/m^2^)	0.74 (0.29–1.85, 0.52)	1.13 (0.53–2.41, 0.75)	0.45
Baseline BMI (≥ 30 kg/m^2^ vs < 25 kg/m^2^)	0.30 (0.10–0.93, 0.04)	0.70 (0.34–1.46, 0.34)	0.29
Treatment-Emergent Decline in Physical Function	1.39 (0.53–3.62, 0.51)	0.36 (0.16–0.83, 0.02)	0.05
Treatment-Emergent Endocrine Symptoms	0.97 (0.45–2.07, 0.93)	2.05 (1.10–3.82, 0.02)	0.11
Treatment-Emergent Pain Interference	2.33 (0.89–6.09, 0.09)	0.54 (0.23–1.25, 0.15)	0.03
Treatment-Emergent Fatigue	2.08 (0.91–4.78, 0.09)	1.51 (0.79–2.86, 0.21)	0.62
Treatment-Emergent Depression	2.07 (0.90–4.79, 0.09)	1.00 (0.51–1.93, 0.99)	0.32
Treatment-Emergent Anxiety	0.76 (0.32–1.82, 0.54)	1.18 (0.63–2.23, 0.61)	0.47
Treatment-Emergent Sleep Disturbance	0.99 (0.45–2.15, 0.97)	1.16 (0.61–2.22, 0.65)	0.68
Multivariate Logistic Regression Model			
AET (AI vs Tam)	2.80 (0.90–8.77, 0.08)	––	––
Mastectomy	2.06 (0.89–4.77, 0.09)	––-	––
Treatment-Emergent Pain Interference	2.49 (0.99–6.24, 0.05)	––-	––
Race (White vs Black/Other)	7.13 (1.29–39.4, 0.02)	2.29 (0.82–6.37, 0.11)	––
Baseline BMI (25–29.9 kg/m^2^ vs < 25 kg/m^2^)	––	0.99 (0.45–2.19, 0.98)	––
Baseline BMI (≥ 30 kg/m^2^ vs < 25 kg/m^2^)	––	0.56 (0.27–1.18, 0.13)	––
Treatment-Emergent Endocrine Symptoms	––	2.86 (1.55–5.26, < 0.001)	––
Treatment-Emergent Decline in Physical Function	––	0.30 (0.13–0.68, 0.004)	––
Higher Stage	––	2.25 (1.52–3.34, < 0.001)	––

Treatment-Emergent symptoms were defined as worsening PRO scores meeting or exceeding the MID at 3 and/or 6 months compared to baseline; AET = Adjuvant Endocrine Therapy, AI = Aromatase Inhibitor, Tam = Tamoxifen, BMI = Body Mass Index, NP = Neighborhood Poverty

a. Odds ratios estimated from logistic regression models estimated with GEE with weight gain status as the dependent variable and terms for each variable and time point

b**.** P-value for interaction between menopausal status and the variable on weight gain status, estimated from logistic regression models estimated with GEE with weight gain status as the dependent variable and terms for menopause status, the variable shown, and their interaction

c. NP rate is the percentage of persons living in a zip code with a family income below the federal poverty line based on United States census data

Different variables were selected for the final multivariate models of factors associated with clinically significant weight gain among pre-and post-menopausal participants. In the final pre-menopausal model, receipt of AI as opposed to tamoxifen (OR 2.8, 95% CI 0.9–8.77, *p* = 0.08), prior mastectomy (OR 2.06, 95% CI 0.89–4.77, *p* = 0.09), treatment-emergent pain interference (OR 2.49, 95% CI 0.99–6.24, *p* = 0.05) and White race (OR 7.13, 95% CI 1.29–39.38, *p* = 0.02) were associated with greater likelihood of weight gain ≥ 5% compared to baseline. In the final post-menopausal model, treatment-emergent endocrine symptoms (OR 2.86, 95% CI 1.55–5.26, *p* < 0.001), higher stage (OR 2.25, CI 1.52–3.34, *p* < 0.001), and White race (OR 2.29, 95% CI 0.82–6.37, *p* = 0.11) were associated with greater likelihood of ≥ 5% weight gain compared to baseline while treatment-emergent decline in physical function (OR 0.3, 95% CI 0.13–0.68, *p* = 0.004) was associated with lower likelihood of ≥ 5% weight gain compared to baseline. In addition, compared to post-menopausal participants with baseline BMI < 25 kg/m^2^, those with baseline BMI ≥ 30 kg/m^2^ were less likely to gain ≥ 5% weight compared to baseline (OR 0.56, 95% CI 0.27–1.18, *p* = 0.13). In our sensitivity analysis, after excluding the type of AET as a candidate variable for the model for pre-menopausal participants, the model selected included the same variables with similar effect sizes (Supplemental Table 5).

## Discussion

In this study, we confirmed that clinically significant weight gain, defined as ≥ 5% compared to baseline, is an important problem, with a cumulative incidence by 5 years after AET initiation of 52% in our study population. While weight gain began soon after initiation of AET for some participants, it occurred later for others. Although pre-menopausal participants were *less* likely than post-menopausal participants to have BMI ≥ 25 kg/m2 at baseline, they were *more* likely to gain ≥ 5% weight by 60 months (67% versus 43%). An additional key finding of our study is that early treatment-emergent symptoms, defined as changes in PRO scores compared to baseline at the 3 and/or 6-month time points that were in the direction indicative of worsening symptoms and that met or exceeded the MID, were frequent, and, in multivariable modeling, were associated with clinically significant weight gain through 5 years of AET in both pre- and post-menopausal participants.

While previous literature has consistently demonstrated an association between adjuvant chemotherapy and weight gain, findings with regard to whether AET is associated with weight gain have been variable across prior studies [6, 7, 13, 17, 37, 41–43, 89]. Our study, in which the majority of participants did not receive chemotherapy, confirms that weight gain during AET itself is a clinically important problem, although the observational design precludes determination of causality. The mechanisms behind weight gain during adjuvant therapy for breast cancer are not clearly defined, but may include factors such as reduced physical activity, dietary changes, increased insulin resistance and inflammation [4, 41, 89, 90]. Additionally, treatment-induced menopause, a transition that may impact physiologic fat accumulation and body composition, may explain the greater weight gain we and others have observed among pre-menopausal compared to post-menopausal women [2, 7–9, 13, 15, 18, 19, 91–94]. Furthermore, young women may be at particular risk for weight gain during adjuvant therapy because responsibilities like career and family may take time away from exercise and healthy eating [11].

A novel aspect of our study is that we identified *early* patient-reported treatment-emergent symptoms (defined as changes in PRO scores compared to baseline at the 3 and/or 6-month time points that met or exceeded the MID in the direction indicative of worsening symptomatology), that, in combination with baseline clinicodemographic factors, were associated with clinically significant weight gain over 5 years of AET. Among participants who were pre-menopausal at diagnosis, patient-reported treatment-emergent pain interference was associated with ~ 2.5-fold higher likelihood of clinically significant weight gain. Although associated with weight gain in univariate analysis in the pre-menopausal participants, treatment-emergent fatigue and depression were not selected for the final model. In post-menopausal participants, patient-reported treatment-emergent endocrine symptoms were associated with 2.86 times higher likelihood of ≥ 5% weight gain while patient-reported treatment-emergent decline in physical function was associated with a 70% lower likelihood of clinically significant weight gain.

Few prior studies have evaluated associations between patient-reported symptoms during AET and weight gain. Available data suggest worsening sexual function, physical activity, endocrine symptoms, sleep disturbance, pain, fatigue and anxiety are associated with weight gain [11, 29, 52, 58, 95–98]. Weight gain is also associated with changes in patient-reported function [11, 51]. Additionally, avoidance of weight gain and maintenance of physical activity are associated with reduced patient-reported symptoms, improved function and improved quality of life [55, 99]. Our study builds on this limited data by comprehensively evaluating multiple common AET-associated symptoms in one cohort and demonstrating that *early* treatment-emergent symptoms during AET are associated with weight gain over 5 years of AET. Moreoever, our study demonstrates that the relationship of specific treatment-emergent symptoms with weight gain varies by menopausal status and that MIDs on PRO measures can detect clinically relevant changes in symptom severity early during AET that are associated with weight gain throughout the course of AET. While further research is needed to confirm these associations between early treatment-emergent symptoms and weight gain during AET in pre- and post-menopausal women, our study suggests that treatment-emergent symptoms identified using changes in PRO scores that meet or exceed the MID in the direction indicative of worsening symptoms may be used to identify patients at risk for weight gain during AET.

The mechanisms linking changes in symptoms and weight gain during AET are uncertain. Reduced physical activity due to joint pain while receiving AET may explain the relationship we observed between increased pain interference and weight gain during AET in pre-menopausal participants [11, 29, 95]. The relationship we observed between declining physical function and lower likelihood of weight gain during AET in post-menopausal participants may be attributable to loss of muscle mass in the setting of frailty. Disrupted sleep due to vasomotor symptoms may lead to fatigue, reduced exercise, and weight gain, explaining the relationship we observed between endocrine symptoms and weight gain in post-menopausal participants. Additionally, other endocrine symptoms such as breast tenderness, mood swings and irritability might lead to decreased physical activity and, in turn, to weight gain.

A difference in likelihood of clinically significant weight gain according to race was seen in both pre-menopausal and post-menopausal participants, with > 5% weight gain from baseline more frequent in White participants. Racial differences in weight gain after breast cancer have been mixed in prior studies. [8, 100–103]. It is possible that differences in weight gain during AET by race are attributable to differences in diet, activity or baseline BMI. While statistically significant, interpretation of differences according to race in this analysis should be made with caution since the majority of participants were White.

Among pre-menopausal participants in our study, prior mastectomy and receipt of AI compared to tamoxifen were also associated with weight gain. Weight gain after mastectomy has previously been reported and may be attributable to post-operative upper extremity and chest wall pain, numbness, and decreased range of motion leading to decreased ability to exercise [9, 104]. While pain interference was associated with weight gain in pre-menopausal women, AI receipt was also independently associated with weight gain potentially due to other side effects not captured on the PROMIS pain interference measure, a tool not specific to joint pain, that may lead to reduced physical activity and weight gain [95]. Additionally, in pre-menopausal women, AI therapy requires induction of menopause via either OFS or ablation which can alter metabolism and lead to weight gain [45, 47, 94, 105].

Among the post-menopausal participants in our study, lower BMI at baseline and higher stage were also associated with weight gain. Lower baseline BMI is a known risk factor for weight gain since 5% body weight in a person with a lower BMI is less absolute weight than in someone with a higher BMI [15, 102, 106]. Our study is consistent with other literature showing patients with higher stage breast cancer are at higher risk of weight gain likely due to different treatment regimens, more intense surgery or radiation treatments which could contribute to the risk of weight gain [2, 9, 18, 106].

Several recent trials have evaluated weight gain prevention and weight loss interventions for patients receiving treatment for breast cancer and for breast cancer survivors, with early data supporting efficacy with regard to weight endpoints [107–117]. Longer term follow-up of these studies and ongoing trials such as the Breast Cancer Weight Loss (BWEL) Study (NCT02750826) will determine if weight loss interventions translate into improvements in breast cancer survival outcomes also. Examples of interventions evaluated to date include face-to-face dietary counseling, telephone dietary counseling, physical exercise regimens and cooking classes, with combined dietary and physical exercise interventions exhibiting the most promising results [107–115]. Given the association between treatment-emergent symptoms and weight gain during AET, it is conceivable that symptom management using evidence- based strategies to manage AET-associated symptoms could mitigate weight gain also [50]. However, to successfully implement weight gain prevention strategies during AET, it is critical to identify at risk patients in whom the interventions may be most impactful. Our study indicates that treatment-emergent symptoms, as reported by patients soon after AET initiation, and baseline clinicodemographic variables can identify patients at particular risk for clinically significant weight gain during AET. In the future, this finding may translate into a strategy to identify patients to whom early interventions to prevent weight gain could be targeted. Additionally, this study and our prior publication, in which we reported symptoms over the 5-year course of AET in this cohort, demonstrate that PRO measures can identify clinically meaningful treatment-emergent symptoms during AET, potentially offering the opportunity for enhanced symptom management strategies that may, secondarily, lead to lower likelihood of weight gain [118].

Strengths of our study include use of clinically obtained weight and height assessments (as opposed to patient-reported measures), frequent weight assessment time points, use of a defined threshold for clinically significant weight gain that is supported by prior literature, and longer follow-up than many prior studies that focused on short-term weight gain during AET [2, 9, 11, 12, 15, 17–19, 93]. The large sample size and contemporary real-world population are also strengths of this study supporting generalizability of our findings. Additionally, our use of validated PRO measures assessing common symptoms during AET at baseline and early during the course of AET plus our use of MID values to define PRO score changes that represent clinically meaningful treatment-emergent symptoms are strengths of this study. Furthermore, the fact that we built separate models for the factors associated with clinically significant weight gain in pre- and post-menopausal participants and that we used the QIC model selection approach to build the multivariable models are other strengths of this study. The QIC model selection strategy is optimal for use with longitudinal data with repeated measures in individual participants and results in models with overall good correlation structure and fit. It must be stated, however, that the QIC model selection approach is based on model likelihood, thus selection of variables for inclusion in a multivariable model is not driven by a p-value threshold. As such, the QIC model selection approach can yield final models in which variables with *p* > 0.05 are included as occurred in this study [86]. However, the directionality of the odds ratios in the final models we present supports the relationships between the selected variables and clinically significant weight gain despite some p-values exceeding 0.05.

Our study also has weaknesses. Given that it was performed at a single institution in the United States with a predominantly White population and that few pre-menopausal participants received OFS and/or an AI, generalizability may be limited. In addition, missing PRO data due to incomplete surveys, reasons for which are unknown, and missing weight data, due to the fact that weight assessments were only available if a participant had a routine clinical visit within the designated windows around PRO time points, are limitations. Furthermore, we did not have information about specific concomitant medications, such as anti-depressants, which can influence weight and may potentially confound the relationship between the variables evaluated and weight gain. We also did not have information about diet, physical activity or other comorbid health conditions, all of which may also be associated with weight gain. Nor did we collect data on other body anthropometrics beyond weight and BMI, such as weight circumference or body fat. Additionally, while the fact that we performed analyses separately according to menopausal status is a strength of our study, it must be noted that we assessed menopausal status at diagnosis and did not capture changes in menopausal status over time and differences in patterns of treatment-emergent symptoms by menopausal status may have largely been driven by differences in receipt of AIs versus tamoxifen in pre- and post-menopausal participants. Further, although a change in a PRO score meeting or exceeding the MID is considered clinically meaningful, it is not certain that a change of this extent is necessarily the *minimal* significant change and MID thresholds may vary in different clinical scenarios [81]. It is possible that, if anchored to weight gain, different MID thresholds on the PRO measures could be identified, strengthening or weakening the associations between early treatment-emergent symptoms and clinically significant weight gain during AET that we identified. Finally, all analyses presented are exploratory and were not pre-specified in the study protocol.

In conclusion, this study confirms that weight gain during AET for early breast cancer is an important clinical problem, especially in pre-menopausal patients. There are almost 4 million breast cancer survivors in the United States and those who are overweight or obese face inferior outcomes with regard to breast cancer, mental health and cardiovascular health [4, 10, 20–28, 30, 31, 34–36, 119]. Strategies to prevent weight gain in breast cancer survivors are an unmet need. We demonstrated that patient-reported treatment-emergent symptoms early during AET and clinicodemographic factors present at AET initiation are associated with weight gain over the course of AET, indicating that patients at risk for clinically significant weight gain can be identified and potentially targeted for weight gain prevention interventions. Future studies should evaluate use of the factors associated with weight gain identified in this study to select pre- and post-menopausal breast cancer patients receiving AET for weight gain prevention interventions, with the ultimate goal of preventing the adverse health effects associated with weight gain and obesity after breast cancer.

## Supplementary information

Below is the link to the electronic supplementary material.

Supplementary file1 (DOCX 18.5 KB)

## Data Availability

The datasets generated during and analyzed during the current study are available from the corresponding author on reasonable request.
