# Supplementary material for: Factors associated with weight gain in pre- and post-menopausal women receiving adjuvant endocrine therapy for breast cancer
Source: J Cancer Surviv. 2023 Jun 1;18(5):1683–96. doi: 10.1007/s11764-023-01408-y (PMC11424737; doi:10.1007/s11764-023-01408-y)
Supplement: Supplementary file 1 — Supplementary file1 (DOCX 18.5 KB) [file 11764_2023_1408_MOESM1_ESM.docx]

**Supplemental Table 1: Baseline Characteristics of Cohort Participants According to Whether They Were Included in Secondary Weight Gain Analysis^a^**

| **Characteristic** | **Included**  **N=309** | **Excluded**  **N=12** |
| --- | --- | --- |
| Mean Age in years (SD) | 62.5 (11.1) | 62.4 (7.8) |
| Race - N (%) | | |
| Black | 32 (10.4) | 1 (8.3) |
| White | 259 (83.8) | 9 (75) |
| Other | 18 (5.8) | 2 (16.7) |
| Endocrine Therapy - N (%) | | |
| Tamoxifen +/- OFS | 132 (42.7) | 8 (66.7) |
| AI +/- OFS | 177 (57.3) | 4 (33.3) |
| Enrolled upon switching from one endocrine therapy to another | 5(1.6) | 1(8.3) |
| Stage - N (%) | | |
| 0 | 25 (8.1) | 3 (25) |
| I | 186 (60.2) | 5 (41.7) |
| II | 77 (24.9) | 2 (16.7) |
| III | 21 (6.8) | 2 (16.7) |
| ER-positive - N (%)^b^ | 309 (100) | 11 (100) |
| PR-Positive - N (%)^b^ | 272 (88.3) | 10 (100) |
| HER-2-Positive - N (%)^b^ | 25 (8.8) | 1 (11.1) |
| Mastectomy - N (%) | 140 (45.3) | 3 (25) |
| Radiation - N (%) | 205 (66.3) | 10 (83.3) |
| Chemotherapy - N (%)^b^ | 86 (28) | 4 (33.3) |
| Mean (SD) Baseline BMI (kg/m2) | 27.5 (5.8) | 32.7 (14.8) |
| Obese – N (%)^b^ | 100 (32.3) | 2 (50) |
| Overweight – N (%)^b^ | 91 (29.4) | 0 (0) |
| Median Number of Concomitant Medications (Range) | 4 (0,29) | 3.5 (0,9) |
| NP Rate^b,c^ >15% - N (%) | 42 (13.7) | 3 (25) |
| Median Follow-up time in Months (Range) | 56.0 (6.9-87.7) | 61.1 (11.5-84.9) |

SD=Standard Deviation, OFS = Ovarian Function Suppression, ER = estrogen receptor, PR = progesterone receptor, HER2 = Human Epidermal Growth Factor Receptor-2, BMI = body mass index, NP=neighborhood poverty

a. Only participants for whom baseline and at least one follow-up weight assessment were available were included in the secondary weight gain analysis.

b. Denominator for percentages was based on the number of known assessments. ER status was missing for one participant excluded from the analysis. PR status was missing for two participants excluded and one participant included in this analysis. HER2 status was missing for 3 participants excluded and 25 participants included in this analysis. Prior chemotherapy was missing for 2 participants included in this analysis. Baseline BMI was missing for 8 participants excluded from this analysis. NP rate was missing for 2 participants included in this analysis.

c. NP rate is the percentage of persons living in a zip code with a family income below the federal poverty line based on United States census data

**Supplemental Table 2: Distribution of Baseline BMI Groups According to Menopausal Status**

| **BMI (kg/m2) Category** | **Pre-Menopausal**  **N (%)** | **Post-Menopausal N (%)** | **p-value^a^** |
| --- | --- | --- | --- |
| Underweight  (<18.5 kg/m^2^) | 5 (5.1) | 2 (1) | <0.001 |
| Normal weight  (18.5-24.9 kg/m^2^) | 47 (47.5) | 64 (30.5) |  |
| Overweight  (25-29.9 kg/m^2^) | 27 (27.2) | 64 (30.5) |  |
| Obese  (≥30 kg/m^2^) | 20 (20.2) | 80 (38.1) |  |

BMI = Body Mass Index

a. p-value for Fisher’s Exact test

**Supplemental Table 3: Number of Participants for whom Weight was Available at Each Follow-up Time Point According to Menopausal Status**

| **Time Point** | **Pre-Menopausal**  **N=99** | **Post-Menopausal**  **N=210** |
| --- | --- | --- |
| 3 months, N (%) | 83 (83.8) | 175 (83.3) |
| 6 months, N (%) | 81 (81.8) | 158 (75.2) |
| 12 months, N (%) | 79 (79.8) | 151 (71.9) |
| 24 months, N (%) | 65 (65.7) | 125 (59.5) |
| 36 months, N (%) | 50 (50.5) | 112 (53.3) |
| 38 months, N (%) | 49 (49.5) | 106 (50.5) |
| 60 months, N (%) | 39 (39.4) | 70 (33.3) |

**Supplemental Table 4: Mean Scores on Patient-Reported Outcome Measures at Each Time Point**

| Domain | Baseline  N^a^  Mean (SD) | | | 3 months  N^a^  Mean (SD) | | | 6 months  N^a^  Mean (SD) | | |
| --- | --- | --- | --- | --- | --- | --- | --- | --- | --- |
|  | Pre-  Menopausal | Post-  Menopausal | All | Pre-  Menopausal | Post-  Menopausal | All | Pre-  Menopausal | Post-  Menopausal | All |
| Physical Function | 98  52.3 (8.7) | 210  50.5 (8) | 308  51.1 (8.3) | 87  53.2 (7.7) | 185  51.2 (8.2) | 272  51.9 (8.1) | 76  54.9 (7.2) | 167  52.5 (8.1) | 243  53.3 (7.9) |
| Endocrine Symptoms | 97  65.6 (7.8) | 210  64.8 (9.1) | 307  65.1 (8.7) | 87  63.5 (8.2) | 185  63.6 (9.1) | 272  63.6 (8.8) | 76  62.7 (8.4) | 167  64.1 (8.3) | 243  63.7 (8.3) |
| Depression | 98  47 (7.6) | 209  44.8 (7.9) | 307  45.5 (7.9) | 86  46.3 (8.2) | 185  4.3 (8.1) | 271  45.0 (8.2) | 76  45.6 (7.4) | 165  44.2 (8.3) | 241  44.7 (8.0) |
| Anxiety | 98  51.8 (9.4) | 210  47.8 (9.1) | 308  49.2 (9.4) | 87  51 (8.6) | 184  46.6 (9) | 271  48.1 (9.1) | 76  50 (8.4) | 167  46.3 (8.7) | 243  47.5 (8.7) |
| Sleep Disturbance | 98  49.2 (8.1) | 210  49 (7.9) | 308  49.1 (8.0) | 87  49.9 (8.2) | 185  50 (8.8) | 272  50.0 (8.6) | 76  48.7 (7.6) | 167  49.0 (8.5) | 243  48.9 (8.2) |
| Fatigue | 98  49.1 (7.7) | 209  48.4 (7.9) | 307  48.7 (7.8) | 87  49.2 (7.3) | 185  47.8 (7.8) | 272  48.3 (7.6) | 76  48.8 (8.4) | 167  47.5 (8.6) | 243  48.0 (8.6) |

SD=standard deviation

a. N at each time point for each domain represents the number of participants who completed the questionnaire at that time point.

**Supplemental Table 5: Multiple Logistic Regression Model of Factors Associated with ≥5% Weight Gain Among Pre-Menopausal Participants Selected after Exclusion of Type of AET from Candidate Variables**

| **Variable** | **Odds Ratio^a^** | **95% CI** | **p-value** |
| --- | --- | --- | --- |
| **Mastectomy** | 2.22 | 0.97-5.09 | 0.059 |
| **Treatment-Emergent Pain Interference** | 2.53 | 0.97-6.61 | 0.057 |
| **Race (White vs Black/Other)** | 7.5 | 1.35-41.63 | 0.021 |

NOTE: Treatment-Emergent symptoms were defined as worsening PRO scores meeting or exceeding the MID at 3 and/or 6 months compared to baseline; AET=Adjuvant Endocrine Therapy

a. Odds ratios estimated from logistic regression models estimated with GEE with weight gain status as the dependent variable and terms for each variable and time point.
